# The Cognitive Impact of the *ANK3* Risk Variant for Bipolar Disorder: Initial Evidence of Selectivity to Signal Detection during Sustained Attention

**DOI:** 10.1371/journal.pone.0016671

**Published:** 2011-01-31

**Authors:** Gaia Ruberto, Evangelos Vassos, Cathryn M. Lewis, Roberto Tatarelli, Paolo Girardi, David Collier, Sophia Frangou

**Affiliations:** 1 Section of Neurobiology of Psychosis, Institute of Psychiatry, King's College London, London, United Kingdom; 2 Social, Genetic and Developmental Psychiatry Centre, Institute of Psychiatry, King's College London, London, United Kingdom; 3 Division of Genetics and Molecular Medicine, King's College London School of Medicine, London, United Kingdom; 4 Department of Psychiatry, Sant'Andrea Hospital, Second Medical School, La Sapienza University, Rome, Italy; Rikagaku Kenkyūsho Brain Science Institute, Japan

## Abstract

**Background:**

Abnormalities in cognition have been reported in patients with Bipolar Disorder (BD) and their first degree relatives, suggesting that susceptibility genes for BD may impact on cognitive processes. Recent genome-wide genetic studies have reported a strong association with BD in a single nucleotide polymorphism (SNP) (rs10994336) within *ANK3*, which codes for Ankyrin 3. This protein is involved in facilitating the propagation of action potentials by regulating the assembly of sodium gated ion channels. Since *ANK3* influences the efficiency of transmission of neuronal impulses, allelic variation in this gene may have widespread cognitive effects. Preclinical data suggest that this may principally apply to sequential signal detection, a core process of sustained attention.

**Methodology/Principal Findings:**

One hundred and eighty-nine individuals of white British descent were genotyped for the *ANK3* rs10994336 polymorphism and received diagnostic interviews and comprehensive neurocognitive assessment of their general intellectual ability, memory, decision making, response inhibition and sustained attention. Participants comprised euthymic BD patients (n = 47), their unaffected first-degree relatives (n = 75) and healthy controls (n = 67). The risk allele T was associated with reduced sensitivity in target detection (p = 0.0004) and increased errors of commission (p = 0.0018) during sustained attention regardless of diagnosis. We found no effect of the *ANK3* genotype on general intellectual ability, memory, decision making and response inhibition.

**Conclusions/Significance:**

Our results suggest that allelic variation in *ANK3* impacts cognitive processes associated with signal detection and this mechanism may relate to risk for BD. However, our results require independent replication and confirmation that ANK3 (rs10994336) is a direct functional variant.

## Introduction

Bipolar Disorder (BD) is a complex, highly heritable disease [1]. A genome-wide association study [2], which combined data from two previously published samples [3] and [4], reported a strong association (p = 9.1×10^−9^) between BD and a region (rs10994336) in *ANK3* on chromosome 10q21. Within the nervous system, the protein coded by *ANK3*, known as Ankyrin3 or AnkyrinG, is localised in the initial axonal segment and the nodes of Ranvier [5]. It is involved in the normal clustering of sodium gated channels thus enabling the propagation of action potentials in myelinated neurons [6].

The focus on the potential relevance of ion channels for psychiatric disorders is relatively new; to date channelopathies were mostly considered in the context of cardiac [7] and motor [8] syndromes and of seizure or migraine disorders [9]. However there is empirical support for the significance of ion channels for the clinical symptoms of BD; both Lithium [10], [11] and antiepileptics that have long been successfully used for the treatment of BD are now known to modulate ion channels [12].

In parallel to developments in genetic studies, the cognitive function of BD patients and their relatives has received much attention, particularly as cognition is considered more directly linked to genetic liability [13]. Evidence from multiple studies [14], [15], [16] support the role of several cognitive processes as potential endophenotypes for BD. Therefore examination of the effect of recently identified risk genes on cognition may prove fruitful in delineating cognitive processes, and by inference neural systems, that may mediate genetic risk.

At present, our understanding of the functional significance of the allelic variation of *ANK3* for complex behaviour, either in health or in disease, is rudimentary. However, it has long been known that retinal ganglion cells are dependent on sodium channels for action potential generation [17]; furthermore, these channels appear to improve contrast sensitivity and signal-to-noise ratio thus enhancing visual information processing [18]. Preclinical and computational models suggest that stimulus encoding within neuronal populations is dependent on the speed of action potential onset [19]. This is determined by the clustering and cooperative activation of voltage-gated sodium channels [20], for which Ankyrin3 is required [6]. Finally, attentional processes are associated with functional coupling of prefrontal and visual cortices within the gamma frequency band [21] which is also dependent on optimal sodium channel function [22]. Therefore investigating the potential effect of *ANK3* on visual sustained attention represents a logical first step in unravelling genotype-phenotype relationships.

To this aim we evaluated cognitive function in patients with BD, their first degree relatives and healthy controls genotyped for *ANK3* and undertook two analyses. A hypothesis led analysis on the effect of the *ANK3* genotype and its interaction with diagnosis on target detection as assessed with the Degraded Symbol Continuous Performance task (DS-CPT) [23], [24]. As understanding of the functional impact of *ANK3* on cognition is currently limited we also undertook a hypothesis generating analyses on the effect of allelic variation in *ANK3* on other aspects of cognition.

## Methods

We genotyped participants of the Vulnerability to Bipolar Disorder Study (n = 189) [25]. Cognitive and clinical information on this sample was already available. Patients were identified by clinicians' referrals and were included if they (a) were aged between 17–65 years (b) fulfilled Diagnostic and Statistical Manual of Mental Disorders, 4th edition, revised (DSM-IV) [26] criteria for Bipolar Disorder, type I (BD-I), (c) had at least one first degree relative unaffected by BD and (d) no family history (up to second degree) of schizophrenia or schizophrenia spectrum disorders. Their siblings and offspring were invited to participate, with the patients' consent, if aged 17–65 and without a personal history of Bipolar Spectrum Disorders.

Healthy volunteers were recruited through advertisement in the local press and were enrolled if they were (a) aged 17–65 years and (b) had no personal or family history of any Axis I or II DSM-IV disorder. Healthy volunteers were selected so that they matched both patients and relatives in gender and level of education. Level of education was rated on a 5-point scale ranging from 1 (no educational qualification) to 5 (post graduate university level qualifications).

Exclusion criteria for the entire sample (patients, relatives and controls) included (a) head trauma resulting in loss of consciousness, (b) personal history of neurological or medical disorders, (c) family history of hereditary neurological disorders and (d) fulfilling DSM-IV criteria for lifetime drug or alcohol dependence and drug or alcohol abuse in the preceding six months.

### Ethics Statement

The study was approved by the Ethics Committee of the Institute of Psychiatry. Written informed consent was obtained from all participants and all clinical investigations were conducted according to the principles expressed in the Declaration of Helsinki.

### Clinical Assessment

All participants were interviewed personally by trained psychiatrists, who were initially blind to diagnostic but not family status (BD family member or unrelated control), using the Structured Clinical Interview for DSM-IV (SCID) for Axis I (patient and non-patient version) [27], [28]. Inter-rater reliability was kappa>0.92 for both instruments. Where applicable, further information about age of onset, number and polarity of previous episodes, number of hospital admissions and current medication (type, dose and duration) was collected from medical notes. Family history of psychiatric disorders was assessed using the Family Interview for Genetic Studies (FIGS) [29] supplemented by medical notes as necessary. Participants were rated using the Hamilton Depression Rating Scale (HDRS) [30], the Young Mania Rating Scale (YMRS) [31], and the Brief Psychiatric Rating Scale (BPRS) [32]. Prior to cognitive assessment all participants with an Axis I disorder were assessed weekly over a period of one month to ensure that at each assessment they (a) scored below 7 in the HDRS and YMRS and (b) there had been no change to their type and dose of medication.

### Cognitive Assessment

All participants were assessed using the same battery administered in a single session by qualified psychologists. An estimate of current full-scale intelligence quotient (FSIQ) was obtained using the Wechsler Adult Intelligence Scale-Revised (WAIS-R) [33]. Sustained attention was measured using the Degraded Symbol Continuous Performance task (DS-CPT) [23], [24]. This task provides a sensitive measure of subtle deficits in signal detection over sustained periods of visual monitoring with minimal working memory involvement. Participants were shown images of numerals degraded to a fixed degree by blurring and superimposing a random pattern of visual noise. The stimuli were presented for 28 ms each at a rate of 1 per second on a computer screen situated 1 meter away from the participants. They were instructed to respond as quickly as possible by button press when a predesignated target numeral “0” appeared. The target numeral occurred in a quasi-random fashion in 25% of the 480 trials. The numerals immediately preceding the target were balanced to eliminate sequence recognition. Although stimuli are presented continuously the data was analysed in blocks of trials. The first 160 trials are practice trials and were excluded from the analysis, while the remainder were divided in 4 blocks of 80 trials each. For each block the following four dependent variables were used: 1) hit rate or number of correct responses, 2) false alarm rate or number of errors of commission 3) response criterion, which reflects the amount of perceptual evidence required prior to responding to a stimulus as a target, and 4) d′, a non-parametric signal detection index of sensitivity, a measure of target detection.

The effect of allelic variation in *ANK3* on any aspect of cognition is not known; given the role of *ANK3* in fundamental aspects of neuronal firing and neural connectivity we explored the possibility that its impact on cognition might be quite broad. Therefore we examined the effect of the genotype on all cognitive tests for which complete information was available for all participants. These were the Stroop Colour Word Test (SCWT) [34], the Wisconsin Card Sorting Test (WCST) [35], a test of rule discovery and perseveration, the Wechsler Memory Scale- 3rd Edition UK (WMS-III) [36], and the Iowa Gambling Task (IGT) [37] which is widely used to assess decision-making. We used the paper and pencil version of the SCWT in which participants are asked to read colour words (non-interference condition) or to name the font colour of incongruous colour words (interference condition). The outcome variable included in the analysis was total correct responses in the incongruent condition. In the WCST, participants were required to sort a deck of cards on the basis of a series of unknown categories. Feedback was given after each match to enable identification of the correct matching rule. After a number of correct responses, the card-sorting category changed. The dependent variables used were number of categories achieved (a measure of rule discovery) and number of perseverative errors. The WMS-III focuses on immediate, delayed and working memory, tested in two modalities, auditory and visual and in two task formats, recall and recognition. Six outcome variables from the test were considered auditory and visual immediate memory, immediate memory, auditory and visual delayed memory and auditory recognition delayed memory. Finally, in the IGT, participants were instructed to maximise simulated monetary gains by selecting a total of 100 cards (one at a time) from four decks (A,B,C, D) displayed on a computer screen. The reward or penalty associated with their choice was displayed on the screen after card selection. Decks A and B were “disadvantageous” as they were associated with large rewards but higher penalties; in contrast, Decks C and D were “advantageous” despite lower gains per card as their penalties were also lower. Participants were not told the distribution of wins and losses associated with the decks; rewards and punishments were sequenced so that it was difficult to work this out. It is expected that over time participants will learn to favour the advantageous cards. Emotional learning is a measure of this derived by subtracting the quotient C+D/A+B of the last 20 trials from that of the first 20 trials.

### Genotyping

DNA was obtained from buccal swabs using established procedures [38]. The *ANK3* T/C (rs10994336) genotype was determined by a ‘TaqMan’ allelic discrimination assay (Applied Biosystems, Assay ID C_31344821_10). End point analysis was performed using the Applied Biosystems 7900HT Fast Real-Time PCR System and genotypes were called with the SDS 2.3 software.

### Statistical analysis

To compare demographic features, we conducted univariate analyses of variance (ANOVAs) for continuous variables with genotype and affection status (BD patient, relative, control) as fixed factors and chi-square tests for dichotomous variables. We used the same approach when comparing the effect of genotype on the clinical characteristics of BD patients.

We examined the normality of distribution of the neurocognitive variables in the entire sample with goodness of fit Kolmogorov-Smirnov tests. For normally distributed variables we used univariate, multivariate or repeated measures analysis of covariance with total BPRS score as covariate to examine the main effects and interaction of genotype and affection status. Although YMRS and HAMD data on the entire sample were available these scales are not standardised for use in non-clinical populations. Therefore, the 24-item BPRS was chosen to allow meaningful comparisons between patient and non-patient groups as it can be used to rate both non-pathological (item ratings below 3) and pathological experiences (item ratings of 4–7).

For non-normally distributed variables we employed a simulation method, whereby we retained the phenotypic data (cognitive variables and affection status) and permuted the observed genotype among individuals using the R statistical package (http://cran.r-project.org). We analyzed the data using the ANOVA and retained the test statistic (F-values). The random assignment of genotypes was repeated 100,000 times to derive a distribution of F-values. The latter analysis was conducted twice; once including only completely asymptomatic participants (BPRS score<27) and once including the entire sample while co-varying for BPRS score. Comparison of the observed F-values with the distribution from the simulation method produced empirical p-values which are robust to the non-normal distribution of the phenotype.

As this is the first study to report on the effect of *ANK3* T/C on neurocognitive task performance *a priori* power calculations were not possible. We therefore calculated Cohen's d′ effect sizes [39] and conducted post-hoc power calculations for the main effect of genotype on all neurocognitive variables using Quanto software (hydra.usc.edu/GxE) assuming a minimum allele frequency 0.067 (hapmap.ncbi.nlm.nih.gov/cgi-perl/snp_details_B36?name=rs10994336&source=hapmap24_B36) and an additive model.

## Results

### Sample Characteristics

**Figure 1:**
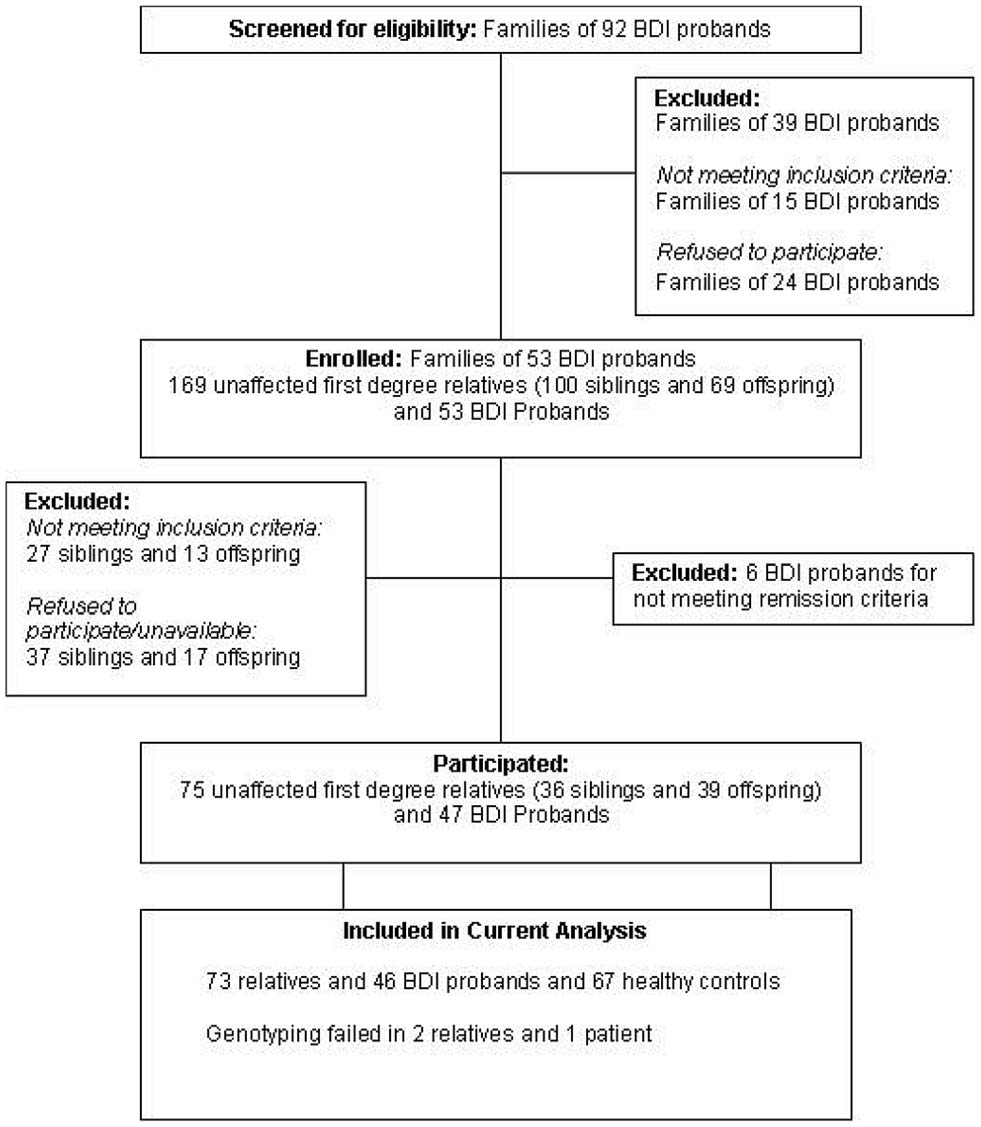
VIBES Sample Identification and recruitment.

Ninety-two BD patients from an equal number of families were screened by telephone interview for eligibility by a trained psychiatrist. Families of 53 BD patients were enrolled in the study; these yielded 47 BD-I patients (6 patients were further excluded as they did not meet remission criteria throughout the study period) and 75 first-degree relatives. All participants were of self-reported British white ancestry. For the current analysis, 1 BD patient and 2 relatives were excluded due to failure in genotyping. Detailed flow chart of study sample recruitment is shown in Figure 1 while demographic and clinical details are shown in Table 1. BD patients had significantly higher HAMD, YMRS and BPRS scores than relatives and controls (all p<0.001) while the two latter groups did not differ from each other. The scores of the three rating scales were highly correlated (r = 0.73, p = 0.0001).

**Table 1:** Demographic and Clinical details of the sample.

	ANK3	BD Patients N = 46	Relatives N = 73	Controls N = 67
**Age (years)**	CC	43.88 (10.68)	32.94 (11.64)	36.20 (13.17)
	CT and TT	45.43 (11.54)	32.80 (15.34)	37.56 (14.65)
**Age range(years)**	CC	21–58	17–58	18–59
	CT and TT	25–56	18–58	20–58
**Educational Level**	CC	3.60 (0.99)	3.75 (0.87)	3.59 (0.98)
	CT and TT	3.20 (0.82)	3.20 (0.84)	3.64 (1.12)
**BPRS total score**	CC	27.78 (3.77)	24.74 (1.71)	24.24 (0.50)
	CT and TT	24.57 (0.98)	24.40 (0.89)	24.82 (0.98)
**Age of onset (years)**	CC	26.40 (8.86)	___	-----
	CT and TT	23.29 (7.74)	___	----
**Duration of illness (years)**	CC	20.29 (10.23)	___	----
	CT and TT	22.57 (8.98)	___	----
Depressive episodes (n)*	CC	3.83 (2.96)	___	----
	CT and TT	11.33 (14.11)	___	----
Manic episodes (n)*	CC	2.57 (2.54)	___	----
	CT and TT	12.20 (16.19)	___	----

Continuous data shown as mean (sd); BPRS = Brief Psychiatric Rating Scale; HAMD = Hamilton Depression Rating Scale; YMRS = Young Mania Rating Scale; GAF = Global Assessment of Functioning *p<0.006.

### Genotype Distribution

Due to the rarity of the T allele (CEU MAF 0.07; www.hapmap.org), only one TT homozygote was identified and was analysed together with the heterozygotes. Genotype frequencies in unrelated participants were in Hardy-Weinberg equilibrium (p = 0.65). In total, 165 individuals (39 BD patients, 58 relatives and 68 controls) were C homozygotes and 21 (7 BD patients, 5 relatives and 9 controls) carried the risk allele T. There were no affection status by genotype interactions for age at assessment (F_2, 185_ = 0.51, p = 0.60), gender or educational status (all p>0.43).

### Genotype Effect on clinical variables

In the BD patient group, there was no genotype effect on age of onset (F_1, 45_ = 0.90, p = 0.35) or duration of illness (F_1, 45_ = 0.16, p = 0.70) or on the rates of psychosis (x^2^ = 0.42, df = 1, p = 0.53). Although the carriers of the risk allele (T) had more depressive (F_1, 45_ = 7.65, p = 0.009,) and manic episodes (F_1, 45_ = 8.53, p = 0.006).

### The relationship of genotype, affection status and cognition

Details of the neurocognitive outcome variables by affection status and genotype are shown in Table S1 (supplemental material).

### Sustained Attention

None of the outcome variables of the DS-CPT were normally distributed. Logarithmic transformation failed to normalise the distribution. Initial exploration of the impact of genotype and affection status on DS-CPT outcome variables was conducted using repeated measures analysis of variance with blocks 1–4 as within subject factor and affection status and genotype as between-subject factors and BPRS as covariate. Where the sphericity assumption was violated, we report results using the Greenhouse-Geisser correction. There was no interaction between block by affection status or block by genotype or block by affection status and by genotype for Hit Rate (all p>0.26), Response criterion (all p>0.12) and reaction time (all p>0.17). The effect size of the genotype was negligible for Hit Rate (Cohen's d = 0.08) and small for Response Criterion and reaction time (Cohen's d<0.25) so it is unlikely that these results reflect lack of power.

The within subjects main and interaction effects for False Alarm (F_6, 177_ = 0.82, p = 0.55) and Sensitivity (F_4.25, 376.66_ = 0.94, p = 0.47) were not significant. In contrast, the interaction between block and genotype was significant both for False Alarms (F_3, 177_ = 2.64, p = 0.05) and Sensitivity (F_2.12, 376.66_ = 2.57, p = 0.05). However polynomial linear within-subject contrasts revealed substantial 2 way interaction of genotype by block for False Alarms and Sensitivity (p = 0.014 and 0.038 respectively). In addition, the between subject effects of genotype was significant for both False Alarm (F_1,177_ = 9.02, p = 0.003) and Sensitivity (F_1,177_ = 10.45, p = 0.001).

Since parametric analysis of non-parametric data may inflate the likelihood of false positive results we employed a simulation method focusing on the last block where the effect size of the genotype was the largest with Cohen d′ being 0.83 and 0.92 for False Alarms and Sensitivity respectively. The effect of genotype remained significant both for False Alarms and Sensitivity; when excluding participants with a BPRS>27 we obtain empirical p-values of 0.0018 and 0.0004 for False Alarms and Sensitivity and the respective p-values when using BPRS as a covariate were 0.0021 and 0.0008. These p-values also withstand Bonferroni correction for multiple comparisons. In all analyses, the risk allele (T) was associated with poorer performance.

### Exploratory analyses of other cognitive variables

There was no significant main effect of genotype or genotype by affection status interaction on test performance on the WAIS-R, SCWT, WCST, and WMS-III and IGT (all p>0.26) (Table 2). The effect of allelic variation in *ANK3* was negligible for FSIQ (Cohen's d = 0.14), WCST categories achieved and perseverative errors (Cohen's d = 0.05 for both) and IGT (Cohen's d = 0.02). For all WMS-III variables the effect of *ANK3* ranged from negligible to small (Cohen's d range 0.03–0.17) with the exception of the composite index of working memory where a moderate effect size (Cohen's d = 0.35) was noted. Similarly a medium effect size of 0.44 was noted for the SCWT. The risk allele (T) was associated with poorer performance on all of these measures.

**Table 2:** Power calculations for the effect of *ANK3* on cognitive function.

Cognitive variable	N	% variance explained	Number needed for 80% power
**WAIS-R FULL IQ**	183	0.16	4843
**DS-CPT: hit rate**	184	0.05	15630
**DS-CPT: false alarms**	184	7.9	95
**DS-CPT: target sensitivity (d′)**	184	11	67
**DS-CPT: response criterion**	184	0.55	1413
**SCWT**	168	1.67	465
**WCST: categories achieved**	186	0.007	118133
**WCST: perseverative errors**	186	0.1	7592
**WMS-III: auditory immediate memory**	184	0.0003	>10^6^
**WMS-III: visual immediate memory**	184	0.18	4431
**WMS-III immediate memory**	184	0.11	7251
**WMS-III auditory delayed**	184	0.18	4293
**WMS-III visual delayed**	184	0.03	29335
**WMS-III auditory recognition delayed**	184	0.14	5787
**WMS-III general memory**	184	0.03	29921
**WMS-III working memory**	184	1.17	670
**IGT: Emotional Learning**	178	0.04	18162

WAIS-R = Wechsler Adult Intelligence Scale-Revised; DS-CPT = Degraded Symbol Continuous Performance Test; SCWT = Stroop Colour Word Test; WCST = Wisconsin Card Sorting Test; WMS-III = Wechsler Memory Scale-3^rd^ edition; IGT = Iowa Gambling Task.

## Discussion

This is first study to examine the effect of allelic variation in *ANK3* on cognition in BD patients, their relatives and healthy controls. We found that the *ANK3* genotype may be specifically associated with visual sustained attention but not with measures of global intellectual function such as IQ. The effect of the *ANK3* genotype was also explored in the domains of memory, response inhibition, decision making and executive function. With the exception of response inhibition the effect sizes observed in all other cognitive domains ranged from negligible to small and are therefore of doubtful biological significance [39].

The version of the CPT employed here depends on the effortful, sequential processing of multiple ambiguous visual targets but not on working memory processes that rely on representations of preceding stimuli [24], [40]. Performance decrements in this task may reflect shifts in sensitivity or response criterion or both. In this study, the risk allele (T) was associated with worse performance in the DS-CPT in terms of False Alarms and Sensitivity but not Hit Rate or Response Criterion. This suggests a specific effect of the *ANK3* in decreasing signal detection rather than lowering response criteria. The latter is often assumed to reflect increased “impulsivity” in responding [41] which does not appear to be the case here; this finding resonates with previous results from independent samples of BD patients suggesting that increased impulsivity may not be a trait feature of BD [42]. The mechanism underlying the association between *ANK3* and reduced sensitivity in target detection is beyond the resolution of our methodology. However, the potential impact of *ANK3* on sodium channel clustering and action potential propagation may impact on stimulus encoding and coupling of prefrontal and visual cortices [18]–[22], both key processes relating to signal detection [43].

There are several important methodological considerations. Firstly, there are several genes as well as behavioural, clinical and cognitive phenotypes for BD that could have been examined in this dataset. However, the simultaneous investigation of all possible combinations corrected for multiple comparisons would require very large datasets with the same or comparable measures on all participants. Although ideal, this is currently not feasible. At the same time, exploring the potential effect size of genetic polymorphisms in smaller, well characterised datasets has the potential of informing future collaborative approaches and reducing the number of target phenotypes. Secondly, the study sample comprised of individuals already in our database and was not specifically obtained for examining the effect of rs10994336. We fully appreciate that our sample size is moderate but we believe that to a large extent this is, at present, inevitable, as ANK3 is not a gene that was theoretically predicted as being of relevance to BD. Thirdly, the risk conferring allele is exceedingly rare and was therefore only identified in large samples pooled from different studies. Such pooled samples do not have detailed phenotypic information apart from diagnosis. Therefore we consider this paper as a first step in the process of unravelling the functional significance of the genes identified for BD. Fourthly, given the moderate size of our sample we were rigorous in the statistical approach employed to minimise the likelihood of spurious findings. Additionally, we have provided estimates of effect size for all our analyses that allows further inspection of the statistical properties of the dataset. Fifthly, since there is no information at present on whether the SNP in the *ANK3* gene (rs10994336) is a direct functional variant or a tagging SNP, which may reflect the effect of another, as yet unknown, variant which has a direct functional effect on both risk of BD and visual sustained attention. Finally, decrements in sensitivity during the DS-CPT have been observed in other disorders notably schizophrenia where they seem to play a more important role than in BD [44]. An association between ANK3 and schizophrenia (albeit with a different risk marker in the first intron of ANK3 on 10q21) has already been reported [45] underlying the importance of this gene for psychotic disorders.

In summary, we report a relatively selective effect of the *ANK3* polymorphism on sensitivity to signal detection affecting individual's sustained attention and possibly contributing to the risk for BD. Further investigations are required to confirm the functional variant of *ANK3* and to investigate the electrophysiological implications of genetic variability in this locus.

## Supporting Information

Table S1 Cognitive Task Performance
(DOC)
