# Supplementary material for: The Cognitive Impact of the ANK3 Risk Variant for Bipolar Disorder: Initial Evidence of Selectivity to Signal Detection during Sustained Attention
Source: PLoS One. 2011 Jan 31;6(1):e16671. doi: 10.1371/journal.pone.0016671 (PMC3031622; doi:10.1371/journal.pone.0016671)
Supplement: Table S1 — Cognitive Task Performance (DOC) [file pone.0016671.s001.doc]

**Table S1.** Cognitive Task Performance

| **Cognitive variable Mean (SD)** | ***ANK3*** | **BD Patients n=46** | **Relatives N=73** | **Controls N=67** |
| --- | --- | --- | --- | --- |
| **WAIS-R: FULL IQ** | CC | 117.71 (18.07) | 115.44 (16.76) | 121.79 (20.02) |
|  | CT and TT | 121.71 (16.27) | 114.4 (16.04) | 123.22 (17.24) |
| **DS-CPT: Hit Rate** | CC | 19.03 (1.91) | 19.12 (3.26) | 19 (2.54) |
|  | CT and TT | 16.83 (4.75) | 19.6 (0.89) | 19.78 (0.44) |
| **DS-CPT: False Alarms** | CC | 0.31 (0.66) | 0.13 (0.34) | 0.27 (0.83) |
|  | CT and TT | 1.33 (1.63) | 0.8 (0.84) | 0.44 (0.73) |
| **DS-CPT: Sensitivity** | CC | 3.69 (2.35) | 4.15 (1.8) | 4.14 (2.02) |
|  | CT and TT | 1.33 (3.83) | 1.2 (3.83) | 2.89 (3.37) |
| **DS-CPT Response Criterion** | CC | 1 (0.02) | 1 (0) | 0.99 (0.06) |
|  | CT and TT | 0.95 (0.05) | 1 (0) | 1 (0) |
| **SCWT** | CC | 44.63 (10.35) | 51.2 (10.35) | 54.62 (10.28) |
|  | CT and TT | 37.83 (11.2) | 48.4 (9.13) | 50.89 (11.25) |
| **WCST: Categories achieved** | CC | 4.67 (1.72) | 5.44 (1.06) | 5.47 (0.99) |
|  | CT and TT | 5.14 (1.46) | 4.6 (1.67) | 5.88 (0.33) |
| **WCST: Perseverative errors** | CC | 2.41 (2.3) | 2.09 (2.31) | 1.7 (1.95) |
|  | CT and TT | 3 (2.71) | 2.2 (3.35) | 1.44 (1.24) |
| **WMS-III: auditory immediate** | CC | 108.03 (16.95) | 113.74 (16.87) | 119.31 (15.48) |
|  | CT and TT | 103.71 (8.65) | 115.4 (10.14) | 119.89 (19.14) |
| **WMS-III: visual immediate** | CC | 107.59 (20.56) | 110.28 (16.88) | 119.59 (19.8) |
|  | CT and TT | 104.14 (17.03) | 106.6 (11.5) | 115.89 (18.74) |

**Table 2 Cognitive Task Performance (continued)**

| **Cognitive variable Mean (SD)** | ***ANK3*** | **BD Patients n=46** | **Relatives N=73** | **Controls N=67** |
| --- | --- | --- | --- | --- |
| **WMS-III: immediate memory** | CC | 109.78 (20.37) | 114.59 (17.56) | 123.67 (19.09) |
|  | CT and TT | 104.71 (12.5) | 113.6 (11.95) | 121.33 (21.09) |
| **WMS-III: auditory delayed** | CC | 111.54 (15.6) | 114.4 (15.3) | 122.8 (12.33) |
|  | CT and TT | 105.43 (9.68) | 117.6 (14.67) | 119.44 (19.28) |
| **WMS-III: visual delayed** | CC | 108.73 (18.36) | 111.28 (16.62) | 118.79 (19.55) |
|  | CT and TT | 110.71 (17.07) | 115 (19.52) | 115.89 (21.23) |
| **WMS-III: auditory recognition delayed** | CC | 110 (16.58) | 110.81 (14.29) | 119.05 (13.68) |
|  | CT and TT | 102.86 (18.9) | 108 (11.51) | 121.11 (14.53) |
| **WMS-III: general memory** | CC | 112.57 (18.63) | 115.34 (16.07) | 125.79 (17.02) |
|  | CT and TT | 108.43 (14.2) | 117.8 (18.98) | 123.67 (22.99) |
| **WMS-III: working memory** | CC | 109.46 (21.02) | 112.97 (16.79) | 118.07 (18.08) |
|  | CT and TT | 108.14 (17.38) | 107.2 (11.21) | 107.67 (16.96) |
| **IGT: Emotional Learning** | CC | 4.17 (13.4) | 6.14 (14.28) | 12.66 (11.6) |
|  | CT and TT | 4.66 (12.56) | 9.2 (13.97) | 10.22 (6.74) |

WAIS-R=Wechsler Adult Intelligence Scale-Revised; DS-CPT=Degraded Symbol Continuous Performance Test; SCWT=Stroop Colour Word Test; WCST=Wisconsin Card Sorting Test; WMS-III=Wechsler Memory Scale-3^rd^ edition; IGT=Iowa Gambling Task
